# Brief Mindfulness-Based Intervention for Seniors—An Exploratory Semi-Randomized Examination of Decentering Effects on Cognitive Functions and Psychological Distress

**DOI:** 10.3390/bs15040466

**Published:** 2025-04-03

**Authors:** Ophir Katzenelenbogen, Daniela Aisenberg-Shafran

**Affiliations:** The Dror (Imri) Aloni Center for Health Informatics, Ruppin Academic Center, Emek Hefer 4025000, Israel; danielaa@ruppin.ac.il

**Keywords:** aging, MBIS, decentering, depression, guided imagery, cognitive functions, psychological status

## Abstract

The need for psychological treatment in the community, specifically in times of crisis and for those in isolation, calls for finding suitable interventions, especially for older adults. The present study examined the effect of a short mindfulness-based intervention emphasizing a ’decentering’ component and an equivalent guided-imagery intervention on cognitive and emotional measures in seniors living in the community. Thirty community seniors (*M*_age_ = 74.7) performed either ’decentering’ or matched guided-imagery intervention, or care as usual as a control. The 8-week interventions included weekly 20 min sessions and daily 10 min home practice. Participants underwent a cognitive and emotional assessment before and after the interventions, which included filling out questionnaires and performing the cognitive Simon task. The results showed improvements only for intervention groups: cognitively, reduced response time and improved accuracy rate were found in the Simon task. Emotionally, reported depression levels were decreased and an increase in reported positive relationships was found. Our study, hence, introduces two intervention protocols, with promising positive effects on psychological and cognitive status. This contributes evidence-based treatments, easy to deliver in nursing homes or retirement communities, for improving the life quality of older adults.

## 1. Introduction

Old age is a life stage characterized by enduring many losses, some of which transpire in the individual’s external environment and some internally. Older adults show a decline in cognitive functions (Salthouse, 2016) and increased psychological distress levels (Thorpe et al., 2006). The COVID-19 crisis enforced isolation on older adults in many countries, increasing their psychological distress (Armitage & Nellums, 2020) and omitting their accessibility to treatment and intervention groups (Aisenberg-Shafran et al., 2021). Mindfulness-based stress reduction (Kabat-Zinn, 1990) is a well-studied intervention found to enhance various cognitive functions and address emotional symptoms (Moore & Malinowski, 2009; Grossman et al., 2004). Notwithstanding the growing body of MBSR research, most MBSR studies have addressed young adult populations and not older adults. Furthermore, while prior studies have explored the benefits of general mindfulness practices in older adults, there is limited research specifically isolating decentering as a core mechanism of change in this population. Given that older adults often experience rigid negative self-perceptions and cognitive–emotional entrenchment, decentering may provide a unique avenue for improving well-being and cognitive flexibility.

In the current study, we designed a mindfulness-based intervention suitable for older adults that highlighted a decentering component, and an equivalent guided-imagery intervention, examining effects on cognitive measures and psychological distress. By comparing a decentering-oriented intervention with a structurally similar guided imagery approach, this study allowed us to explore the potential role of decentering in cognitive and emotional functioning in later life. These interventions can be delivered in the community, in nursing homes or assisted living residences, to contribute to the well-being of older adults, even in times of social isolation or limited mobility.

Cognitive decline accompanies aging, whether among healthy or pathological individuals. Manly et al. (Manly et al., 2005) found that 17% of urban adults aged 65 and older were classified as having mild cognitive impairment (MCI) in single or multiple domains. In follow-up studies on similar populations, 2.6–4.8% of the participants with MCI at baseline met dementia criteria within two years (Mitchell & Shiri-Feshki, 2008; Brodaty et al., 2013). Age appears to have differential effects on independent cognitive domains. For instance, working memory task performance decreases with age (West & Alain, 2000; Raz et al., 2005), whereas semantic memory performance is preserved within healthy older adults. Most domains affected by aging tend to be related to executive functions (Grady, 2012). One such domain is conflict monitoring, an element of the process of cognitive control. Cognitive monitoring has been defined as the ability to suppress irrelevant information and engage selective attention (e.g., M. M. Botvinick et al., 2001). Cognitive decline in old age can be observed in various measures, among them the Simon task (Lee et al., 2005; Aisenberg et al., 2014).

The Simon task is a simple experimental paradigm used to assess control processes under speeded conditions. The paradigm typically contains two basic trial types: congruent trials (when the target is presented on the same side as the instructed key-press) and incongruent trials (when the target is presented on the opposite side of the instructed key-press). Response time (RT) is slower for incongruent targets, suggesting that target location inherently biases response-related processes, though it is irrelevant to task demands. This gap between RT for congruent and incongruent trials is termed the Simon effect.

Another key finding regarding the Simon task among young adults is the diminishing or reversal of the Simon effect when the preceding trial is incongruent (termed the *Gratton*: Gratton et al., 1992). This finding has been viewed as a manifestation of the cognitive-control process used in preparation for the presentation of the next stimulus (M. M. Botvinick et al., 2001; Gratton et al., 1992; Wühr & Ansorge, 2005). In other words, inhibition is activated in the incongruent trials to suppress the irrelevant dimension (i.e., the target’s position). This activation contributes to suppressing the same irrelevant dimension in the subsequent incongruent trial (M. M. Botvinick et al., 2001; Kerns et al., 2004; M. Botvinick et al., 1999).

The Simon effect was found to be stronger among old than among young adults (Van der Lubbe & Verleger, 2002), indicating that aging is related to the difficulty in suppressing a task-irrelevant dimension. This leads to slower RT when confronted with stimuli requiring cognitive-control functioning (Aisenberg et al., 2014). Notably, the Gratton effect was not found among old adults (Aisenberg et al., 2014), as they showed a significant Simon effect after both the congruent and incongruent trials. 

Health constrains, occurring naturally in aging, have been identified as a cause for the decline in subjective well-being (Kunzmann et al., 2000). Depression has been associated with frequent visits to medical services, reduced quality of life, and increased suicide risk (Serby & Yu, 2003). A cross-sectional study of 999 people aged 65 and older found 20% of the participants reported psychological distress (Paul et al., 2006). Mood disorders are commonly reported or diagnosed in older adults (Alexopoulos, 2005; Kessler et al., 2012). For example, about 12% of the old age population suffer from anxiety disorders criteria over a 12-month course (Byers et al., 2010).

Mindfulness is a practice inspired by Buddhism, defined as an individual’s nonjudgmental observation of their ongoing experiences (Kabat-Zinn, 1990; Creswell, 2017). Mindfulness has been widely recognized for its role in promoting emotional well-being and cognitive functions across different age groups (Jha et al., 2015; Goldberg et al., 2022; Galante et al., 2023; Zenner et al., 2014). Recent findings suggest that mindfulness is not only a skill developed through practice but also a general disposition that varies among individuals (Kabat-Zinn, 1990; Rau & Williams, 2016). For example, a study by Perilli et al. (2020) found that adolescents with a stronger mindfulness disposition experienced lower levels of depression, anxiety, and anger, alongside greater self-forgiveness. Consequently, it is apparent that mindfulness-related attitudes can be associated with emotional regulation and well-being, even outside the context of formal training. However, mindfulness is not only a natural disposition but also a skill that can be intentionally cultivated through practice to enhance its benefits.

Mindfulness-based stress reduction (MBSR) interventions typically comprise eight weekly 2–2.5 h sessions, accompanied by a daily 45 min home practice session (Crane, 2017). MBSR was found to improve general well-being and mental and physical health among healthy individuals and cognitive functioning among young adults (Moore & Malinowski, 2009; Verhaeghen, 2021). A recent meta-analysis found MBSR to be effective in improving various biopsychosocial conditions, including depression, anxiety, stress, insomnia, addiction, psychosis, pain, hypertension, weight management, and cancer-related symptoms, while also enhancing prosocial behaviors. Its benefits extend across diverse settings, including healthcare, schools, and workplaces (D. Zhang et al., 2021). Even interventions of four daily 20 min sessions without prior experience were found to increase young adults’ ability to sustain attention and improve visuospatial processing, working memory, and executive functions (Zeidan et al., 2010). Addressing emotional aspects, MBSR has been shown to improve symptoms of depression and anxiety in a wide range of severities in a clinical population and regulate anxiety among healthy individuals (Chiesa & Serretti, 2009; Hofmann et al., 2010).

Several studies have examined MBSR interventions among older adults, demonstrating significant reductions in psychological distress, as well as improvements in subjective well-being and cognitive functions such as working memory (Malinowski et al., 2017; M. O’Connor et al., 2014; McHugh et al., 2010). These effects were observed in both healthy seniors and those with clinical symptoms such as PTSD, chronic insomnia, and recurring depression (Young & Baime, 2010; Perez-Blasco et al., 2016; Keller et al., 2014; Kitsumban et al., 2009; Kumar et al., 2014; Moss et al., 2015; Glück et al., 2016; J. X. Zhang et al., 2015; Smith et al., 2007). However, traditional MBSR programs can be demanding due to extensive training and time commitments, which may be challenging for many older adults (Pardo et al., 2007; Marzetti & Leeuwenburgh, 2006).

While various brief interventions have been tested, their findings remain mixed: some show promising outcomes in reducing symptoms of burnout and retaining effectiveness in stress reduction (Mackenzie et al., 2006; Bergen-Cico et al., 2013; Chen et al., 2013; Shearer et al., 2016; Cavanagh et al., 2013), whereas others report null or small effects (e.g., Somaraju et al., 2023; Schumer et al., 2018). Likewise, when focusing on cognitive functioning, results also vary: some studies note improvements in attention and executive functioning (Jankowski & Holas, 2020; Baranski, 2021), while others demonstrate inconsistent or null findings in working memory and inhibitory control (Quek et al., 2021; Hartanto et al., 2023). Thus, it is possible that abbreviating the intervention reduces participants’ exposure to certain mechanisms, highlighting the need to identify which components are most critical for improving outcomes. Notably, while brief mindfulness interventions have been increasingly explored in younger and middle-aged populations, research on their application for older adults remains sparse. Most studies on mindfulness for seniors have focused on full-length MBSR programs (e.g., Malinowski et al., 2017; M. O’Connor et al., 2014), while investigations into the feasibility and effectiveness of shorter interventions in this demographic are limited. This gap is particularly important given the unique cognitive and emotional challenges faced by older adults, highlighting the need for interventions that are both accessible and effective. Recently, we developed an intervention (Aisenberg-Shafran & Harmatz, 2023) designed to improve clinical symptoms and maintain cognitive abilities in this specific older population by emphasizing a potential key mechanism, namely, *decentering.*

Our intervention was based on theoretical studies that sought to identify the mechanisms of change in the MBSR program (Shapiro et al., 2006; Coffey et al., 2010; Vago & Silbersweig, 2012). Various program components have been suggested in light of the intervention’s effects. For example, Hölzel et al. (2011) integrated psychological studies with neuroscientific findings into four components: (a) attention regulation––the ability to notice one’s mind wandering off and bring it back to the chosen object or task; (b) body awareness––the ability to focus attention on an object of internal sensory experiences, such as emotions, body sensations, and breathing; (c) emotion regulation––the ability to shape responses to emotions through regulatory processes (Ochsner & Gross, 2005); (d) change in perspective of the self (also termed decentering/reperceiving)––a mental process of internalization, perceiving the self as an entity that is constantly changing (Olendzki, 2010). Several studies have sought to conceptualize the efficacy of mindfulness-based therapies, mainly through one of these components, such as emotion regulation (Ortner et al., 2007), body awareness (Carmody & Baer, 2008), attention regulation (Moore & Malinowski, 2009; Chan & Woollacott, 2007; Anderson et al., 2007) and decentering (MacLean et al., 2010).

The current study investigated the effect of short mindfulness-based intervention sessions, highlighting the mechanism of decentering and an equivalent more simplistic guided-imagery intervention, on cognitive functions and well-being among older adults. As mindfulness-based interventions include relatively lengthy sessions, time-bound interventions necessitate a careful determination of session content to maximize the benefit for the participants. Understanding the mechanisms and components involved in the intervention and their effectiveness can lead to the design of briefer and simpler interventions that may prove suitable for a wider range of populations. For the current study, we chose the designed mindfulness-based intervention for seniors (MBIS; Aisenberg-Shafran & Harmatz, 2023) with a decentering orientation (D-MBIS) and created a matching guided-imagery protocol for examination.

Decentering is defined as the process in which the individual observes their feelings and thoughts as ephemeral events, with no reactivity, alongside acceptance. This type of thinking, characteristic of meta-awareness, was found to involve executive monitoring, whereby the individual can relate to experiences as events (Carmody et al., 2009). Unlike other mindfulness components that target primarily physiological arousal or specific emotional responses, decentering more fundamentally reorients one’s sense of identity and self-appraisal (Fresco et al., 2007; Vago & Silbersweig, 2012). That is, rather than simply noticing or managing difficult emotional states, decentering teaches individuals to recognize that thoughts and feelings are transient mental events, not fixed truths or reflections of personal worth (Bishop et al., 2004; Lebois et al., 2015). In older adults—who often struggle with age-related role changes, losses, and existential concerns (Malette & Oliver, 2006; Carr & Fang, 2023)—this capacity to “take a step back” helps uncouple negative thought patterns from self-identity (Lebois et al., 2015). By targeting habitual patterns of identification with these thoughts, decentering can profoundly impact entrenched mental habits (e.g., self-criticism) and maladaptive affective cycles (Vago & Silbersweig, 2012). As a result, it may confer deeper benefits than mindfulness skills limited to sensing bodily cues or regulating emotions. Indeed, evidence suggests that increased metacognitive awareness can even help prevent depressive relapses (Teasdale et al., 2002). Moreover, when internal awareness of psychological phenomena develops through mindfulness, participants consistently report more clarity and less identification with negative mental processes (MacLean et al., 2010). Therefore, we found the decentering component particularly relevant avenue for older adults seeking to maintain well-being amid cognitive decline and life transitions.

Attention regulation is needed for maintaining attention to instructions and returning to them when distracted. Also, attention regulation was found necessary for remaining engaged in meditation, a critical mechanism that is often taught early in mindfulness practice (Hölzel et al., 2011). Thus, attention regulation appears to be a keystone of the other subsequently acquired components. Therefore, whereas the current study aimed to examine the decentering component, we anticipated that attention regulation would be difficult to isolate from other intervention elements. Consequently, attention regulation was incorporated into the administered D-MBIS program.

Importantly, to shorten the intervention, in keeping with our focus on the decentering component, other components, such as body awareness and emotion regulation, were intentionally excluded. The effects of decentering exercises were compared with those of the equivalent guided imagery intervention, both compared to same measures during care as usual (control group). The guided imagery was selected as an appropriate comparison in the experimental methodology, as it is a well-established practice for achieving a wide range of health-related outcomes that applies cognitive resources instead of a therapeutic-oriented procedure (Hart, 2008). Whereas both intervention types are administered by an instructor, mindfulness is more open in nature. Both intervention types typically include a pre-formulated script, but mindfulness explicitly and ultimately relies on the participants’ unique experience, thus demanding extensive use of cognitive resources (e.g., Bishop et al., 2004).

We predicted that both mindfulness and guided imagery interventions would be beneficial for participants’ psychological states. However, we postulated that our D-MBIS intervention would be superior to guided imagery in enhancing cognitive functions. Thus, we posited the following hypotheses: Hypothesis 1––Time will interact with group type in all measures, such that the intervention groups, but not the control group, will show the effect of time. (1a) For psychological distress, the passage of time will decrease depressive symptoms and emotional measures. (1b) For cognitive abilities, the passage of time will have a differential effect between the intervention groups, such that the D-MBIS group will incur greater benefits than the guided imagery group. Hypothesis 2––Mindfulness manipulation for D-MBIS will increase mindfulness self-report levels.

## 2. Methods

### 2.1. Trial Design

This was an exploratory, semi-randomized controlled trial using a 2 (Group: D-MBIS, Guided Imagery) × 2 (Time: Pre, Post) factorial design. The study included three arms: a decentering-oriented mindfulness-based intervention (D-MBIS), a structurally matched guided imagery intervention, and a care-as-usual control group. The study interventions comprised 10 meetings. In Sessions 1 and 10, intervention group participants were screened before and after the intervention using the following measurements (further information on each measure is discussed below): MMSE and Simon task to estimate cognitive abilities; Five-Facet Mindfulness Questionnaire (FFMQ), Patient Health Questionnaire (PHQ-9), the Beck Depression Inventory-II (BDI-II), and Psychological Well-Being Scales (PWB) to assess participants’ current mental state. Sessions 2–9 comprised eight weekly half-hour meetings and a daily 10 min home practice over the 8-week program. Control group participants were assessed twice, in Sessions 1 and 10, using three measures: MMSE, the Simon task, and PHQ-9.

#### Ethical Approval and Pre-Registration

This study was conducted according to the guidelines of the Declaration of Helsinki, and approved by the Ethics Committee of Beer Yaakov Mental Health Center (protocol code 579, date of approval 24 October 2017). Although the interventions were conducted in November–December 2017, prior to the widespread adoption of pre-registration practices, the study was later retrospectively registered on the Open Science Framework (OSF; https://doi.org/10.17605/OSF.IO/RHS63). In addition, it was incorporated into a broader research project pre-registered on ClinicalTrials.gov (NCT04165005; 15/11/2019), with a clear declaration that the data had already been collected.

### 2.2. Participants

Thirty-two Israeli participants (25 females), aged 65 and older, were recruited through two community centers in Emek Hefer (See Supplementary Materials for a flow diagram summarizing participant enrollment and attrition) during 2017. Participation in the study was voluntary as part of the enrichment activities offered at the center. Written informed consent was obtained from all participants included in the study. Inclusion criteria comprised membership in the community center, being aged 65 or older, scoring 24 and above in the Mini-Mental State Examination (MMSE; Folstein et al., 1975), and having high-level proficiency in Hebrew. Based on the screening questionnaires, 30 individuals (*M*_age_ = 74.83, *SD* = 4.27) were invited to the study. Half of the participants were assigned to the intervention groups (16) and half to a control group-care as usual. Two women chose to withdraw after completing the prior-intervention questionnaires; hence, the interventions began with 14 participants (12 female).

### 2.3. Interventions

#### 2.3.1. D-MBIS

The instructions for the D-MBIS induction were adapted from the sitting mindfulness meditation exercise used in the MBSR program by Kabat-Zinn (1990): “Observe your mind with moment-to-moment awareness. When attention wanders, note it without judgment and then gently bring awareness back to the breath”. Variants of these instructions were repeated every 30–60 s.

#### 2.3.2. Guided Imagery

The content of the guided imagery was partially adapted to the instructions of the D-MBIS group. Participants were asked to listen to a story about a fictional character with no further intervention. The content of this intervention group was matched to the D-MBIS group content. For example, when the D-MBIS group practiced observation through the technique of “mind as a mountain”, the theme of the guided imagery session was a traveler climbing a mountain. Importantly, in contrast to the D-MBIS instructions, the guided imagery participants were not given any specific guidance about what to do if their mind wanders besides bringing it back.

#### 2.3.3. Control Group

Participants assigned to the control group received care as usual with no intervention between pre- and post-test sessions.

### 2.4. Outcomes

#### 2.4.1. Primary Cognitive Measures

The Simon task: In the visual version of the Simon task used here, a colored target appeared either on the right or the left of a fixation (Simon & Small, 1969). In our paradigm, the targets appeared as circles in four colors (blue, red, yellow, and green), 5° in diameter, and could each appear 13° right, left, above, or below the central fixation (following Aisenberg & Henik, 2012; Aisenberg et al., 2018). Participants were asked to press a left key for two of the colors and a right key for two of the colors (Craft & Simon, 1970; Proctor & Lu, 1994; Umiltà & Nicoletti, 1985). Possible combinations of the above colors and locations yielded four incongruent targets (target appearing on the opposite side of the color-specific response key), four congruent trials (target appearing on the same side of the color-specific response key), and four neutral trials (target appearing below or above the fixation point). Following Aisenberg et al. (2018), our blocks contained 91 trials, with at least 30 trials of each congruency type. The nine possible sequential pairings (e.g., congruent following congruent, incongruent following congruent) between target types were presented in a randomized order, 10 times in each block. Participants completed a 16-trial practice block and two experimental blocks.

Mini-Mental State Examination (MMSE; Folstein et al., 1975): The MMSE examines cognitive abilities to identify cognitive decline and dementia. More specifically, it assesses immediate and short-term memory, orientation, language and praxis, attention and calculation (Lancu & Olmer, 2006). The MMSE demonstrates reasonable inter-observer reliability, with a Cohen’s kappa coefficient of 0.97 (D. W. O’Connor et al., 1989). Moreover, MMSE has relatively low false positive and false negative reliability ratings for brief cognitive screening of older adults (MacKenzie et al., 1996).

#### 2.4.2. Primary Psychological Measures

Beck Depression Inventory-II (BDI-II; Beck et al., 1996): The BDI-II is a widely used 21-item self-report inventory measuring the severity of depression in adolescents and adults. Items are presented on a 4-point Likert-type scale, ranging from 0 (not at all) to 3 (nearly every day), reflecting the severity on each item. Higher total scores indicate more severe depressive symptoms. It appears that the questionnaire has good internal reliability among older adults with an alpha coefficient of 0.91 (Gallagher et al., 1982).

The Patient Health Questionnaire-9 (PHQ-9; Kroenke & Spitzer, 2002): The PHQ-9 is the depression module from a self-reported diagnostic instrument for screening common mental disorders. Items are presented on a 4-point Likert-type scale, ranging from 0 (not at all) to 3 (nearly every day). Scores of 10 and above have been found to have a sensitivity of 88% and a specificity of 88% for diagnosing major depression (Kroenke & Spitzer, 2002).

The Ryff Scales of Psychological Well-Being (PWB): The PWB is a 42-item theoretically grounded instrument that focuses on measuring six aspects of psychological well-being, presented on a 6-point Likert-type scale (Ryff & Keyes, 1995). Good internal consistency among six measures has been reported (Bloch-Jorgensen et al., 2018). These included five Cronbach alphas ranging from 0.76 to 0.90 and one measure with a Cronbach alpha of 0.49.

Five-Facet Mindfulness Questionnaire (FFMQ): The FFMQ is a 39-item mindfulness-level self-report measure comprising five factors representing currently conceptualized mindfulness processes (i.e., observing, describing, acting with awareness, non-judging of inner experience, and nonreactivity to inner experience; Baer et al., 2006). Items are presented on a 5-point Likert-type scale. Good internal consistency and reliability for each subscale were found, ranging from 0.82 to 0.91 (Campbell et al., 2017).

### 2.5. Sample Size

A priori power analysis referred to F tests of repeated measures, within–between interaction, with an estimated medium effect size of *f* = 0.25, including six measurements comparing three groups, yielded a required total of 30 participants.

### 2.6. Randomization and Blinding

Due to geographic constraints, participants from Emek Hefer were assigned to the control group, and randomization was only feasible for participants attending the community center, who were randomly assigned to either the D-MBIS or guided imagery groups. Three participants were assigned based on scheduling limitations. The control group was recruited from a separate geographic location and was not randomized. Participants were blinded to their assigned intervention and unaware whether their program was mindfulness-based or guided imagery.

### 2.7. Implementation and Fidelity

Both interventions were delivered by the same trained instructor at the same location and time of day (17:00 for D-MBIS; 17:30 for guided imagery). A master’s student in gerontological–clinical psychology administered the sessions face to face, ensuring fidelity to the protocols and facilitating participant involvement. Session scripts and structure were matched—the instructions for each intervention lasted 20 min. The beginning instructions of the two interventions were matched: “Now, we’re going to do an exercise for 20 min. First, settle into a comfortable sitting position”. Additionally, the two interventions provided identical instructions for what to do if one’s attention wanders off: “bring your mind back” to the focus of the exercise. Participants who missed sessions were offered phone-guided meditations. Home practice was encouraged but not formally monitored. Homework compliance was self-reported but not recorded for fidelity assessment. See Supplementary Materials for additional details of the interventions.

## 3. Results

Participants in both interventions completed their respective programs. Several participants did not attend all meetings (see Table 1). Phone-guided meditation was delivered to every participant after missing a session. A comparison of demographic variables was performed using one-way ANOVA and t-test for independent groups. No significant demographic differences were found between the groups (see Table 1). All 16 control group participants completed both scheduled assessments.

To determine potential pre-intervention between-group differences in all mental state and mindfulness measures (PWB, BDI-II, PHQ-9, and FFMQ), a *t*-test for independent groups was conducted (see Table S2 in the Supplementary Materials). No baseline difference reached significance.

### 3.1. Mindfulness Skills

Change in mindfulness level was measured using the five FFMQ sub-scales. Only the significant effects of the sub-scales are reported below.

#### 3.1.1. Non-Judgment

**Figure 1:**
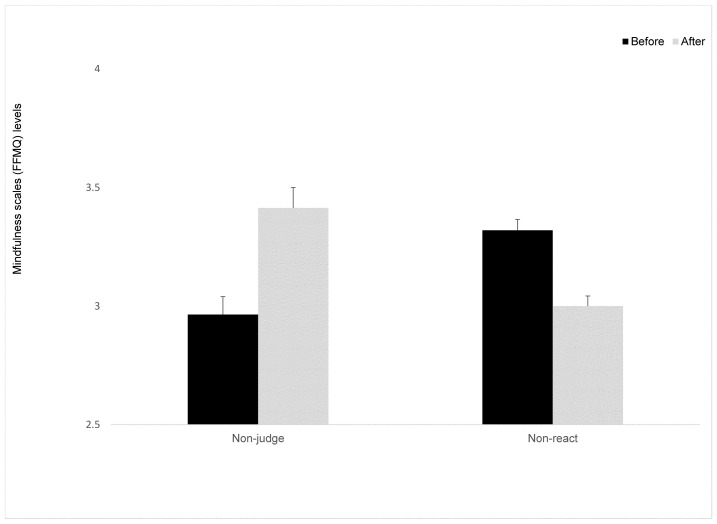
Mindfulness scales (FFMQ) levels at Time 1 and Time 2 for the intervention groups. Means (*SD*). Error bars represent standard error from the mean.

Only ten participants were entered into this part of the statistical analysis. Four participants were excluded from analyses due to a high number of missed items on this scale. A mixed ANOVA was conducted, with time as the within-participant factor. The main effect for time was marginally significant, *F*_(1,8)_ = 7.16, *p* < 0.052, ηp^2^ = 0.47 (see Figure 1), revealing an increase in non-judgment in both intervention groups. The Group X time interaction on non-judgment was not significant, *F*_(1,8)_ < 1. 

#### 3.1.2. Non-React

The change in the level of reaction to external and internal events was measured using the Non-React sub-scale. A mixed ANOVA was conducted with time as the within-participant factor. A marginally significant main effect for time was found, *F*_(1,11)_ = 4.35, *p* < 0.056, ηp^2^ = 0.28 (see Figure 1). Surprisingly, the direction indicated a decreased ability to sustain automatic reactions following the interventions. The Group X time interaction on non-react was not significant, *F*_(1,11)_ < 1.

### 3.2. Depression

**Figure 2:**
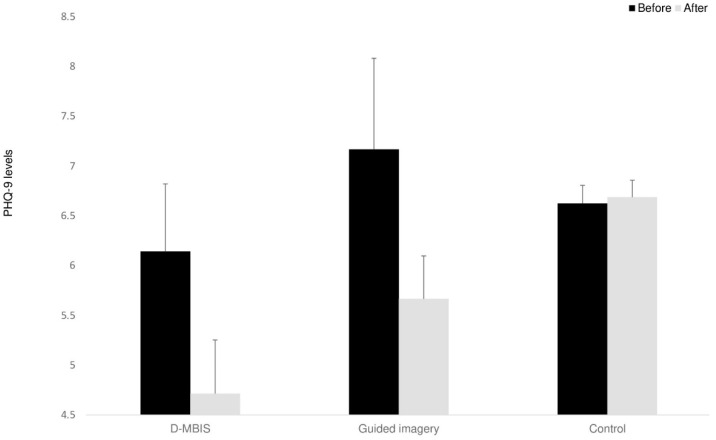
Depression (PHQ-9) levels at Time 1 and Time 2. Means (*SD*). Error bars represent standard error from the mean.

The change in depression level was measured using the BDI-II and PHQ-9 for the two intervention groups and just the PHQ-9 for the control group. One D-MBIS participant did not complete these two questionnaires on the post-intervention measure and was thus excluded from the analysis. A mixed ANOVA was conducted, with time as the within-participant factor. Baseline levels (see Table S2 in the Supplementary Materials) seemed to indicate minimal or mild depression levels (Beck et al., 1996; Kroenke & Spitzer, 2002). According to our hypotheses, PHQ-9 showed reduced depressive symptoms after intervention for both intervention groups, but not for the control group, *F*_(1,26)_ = 4.00, *p* < 0.05, ηp^2^ = 0.13 (see Figure 2). No significant change was observed for the BDI-II scores for the two intervention groups, *F*_(1,11)_ < 1.

### 3.3. Psychological Well-Being

Change in well-being was measured using the six sub-scales of the PWB questionnaire. Only the significant effects of the sub-scales are reported below.

#### Positive Relations

**Figure 3:**
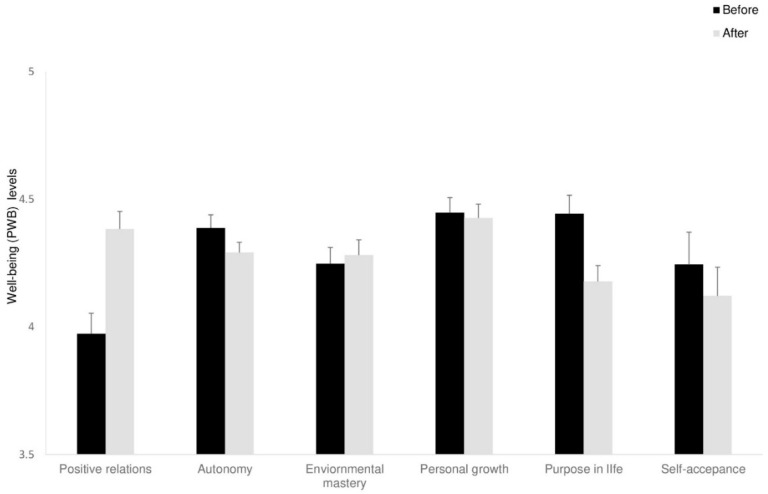
Well-being (PWB) levels at Time 1 and Time 2 for the intervention groups. Means (*SD*). Error bars represent standard error from the mean.

Twelve participants of the intervention groups were entered in this part of the statistical analysis. Two participants were excluded from the analysis due to a high number of missing items. A mixed ANOVA was conducted, with time as the within-participant factor. In line with our hypotheses, a significant main effect for time was found, *F*_(1,10)_ = 28.22, *p* < 0.001, ηp^2^ = 0.74), revealing that enhancement in positive relations was reported for both interventions (see Figure 3). The Group X time interaction on positive relations was also marginally significant, *F*_(1,10)_ = 5.53, *p* < 0.054, ηp^2^ = 0.73), indicating positive relations; the guided imagery group benefited more from the intervention than the D-MBIS group.

### 3.4. The Simon Task

RT for correct responses and accuracy were calculated for each participant in each condition. Responses above 2.5 *SD* or less than 150 ms were excluded from the analysis. Two participants were excluded from the analysis, one due to a technical problem in coding results at Time 1 and one due to low accuracy rates (below chance).

#### 3.4.1. Accuracy Rate

**Figure 4:**
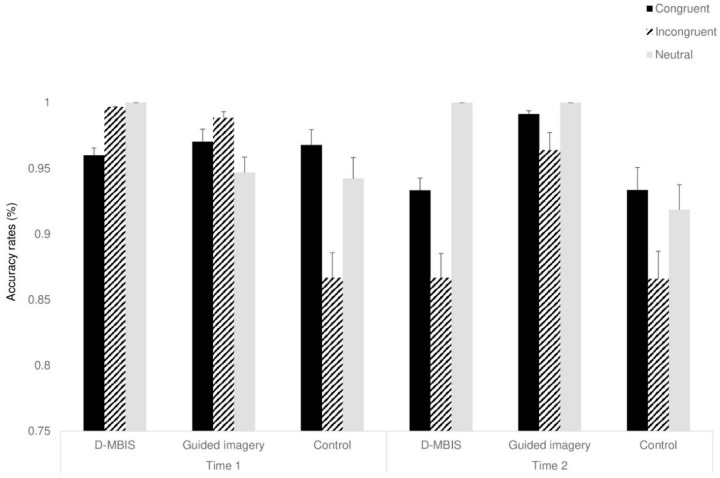
Accuracy rates for the study groups at Time 1 and Time 2. Means (*SD*). Error bars represent standard error from the mean.

A mixed ANOVA, with congruency (congruent, incongruent, neutral) and time (before, after) as within-participant factors and group as between-participant factor, was conducted. A main effect for congruency was found, *F*_(2,48)_ = 3.43, *p* < 0.05, ηp^2^ = 0.12, showing greater accuracy in response to congruent trials than in incongruent trials, *F*_(1,24)_ = 4.60, *p* < 0.054, ηp^2^ = 0.16. The two-way Time X Congruency interaction was marginally significant, *F*_(2,48)_ = 2.64, *p* < 0.058, ηp^2^ = 0.09). Notably, even though the three-way Time X Congruency X Group interaction was not significant, *F*_(4,48)_ = 2.06, *p* < *n.s*, ηp^2^ = 0.14, several effects in line with our hypotheses were observed before and after the interventions: whereas at Time 1, no differences in accuracy rates for either congruent or incongruent trials were observed between the groups, *F*_(1,24)_ < 1 at Time 2, a significant difference in the Simon effect (incongruent–congruent accuracy rates) was found between the two intervention groups and the control group, *F*_(2,48)_ = 14.03, *p* < 0.001, ηp^2^ = 0.36 (see Figure 4). Namely, the intervention groups showed a smaller Simon effect than control group at Time 2. All other effects were not significant (see Table S3 in the Supplementary Materials).

#### 3.4.2. Response Time—RT

A mixed ANOVA, with congruency (congruent, incongruent, neutral), previous trial (congruent, incongruent, neutral), and time (before, after) as the within-participant factors and group as the between-participant factor was conducted. A marginally significant main effect for time was found, F_(1,23)_ = 5.07, *p* < 0.053, ηp^2^ = 0.18, as RTs were faster following the interventions (in line with the expected improvement for the re-test). Moreover, a main effect for congruency was found, F_(1,23)_ = 8.2, *p* < 0.05, ηp^2^ = 0.26, indicating that RT for congruent trials were shorter than for incongruent trials. For simplification, we excluded natural trials from the analysis. Partly in line with our predictions, the three-way Time X Group X Congruency interaction approached significance, F_(2,23)_ = 3.47, *p* < 0.054, ηp^2^ = 0.23, indicating that following the interventions, both intervention groups showed shorter RT than the control group, F_(1,23)_ = 10.83, *p* < 0.05, ηp^2^ = 0.32. Looking at sequential dependencies, we addressed only the intervention groups to simplify the analysis: the two-way interaction between time and previous trial was marginally significant, *F*_(1,8)_ = 6.75, *p* < 0.053, ηp^2^ = 0.45, showing partly as we expected, that RT following incongruent trials improved (was reduced) after the interventions.

**Figure 5:**
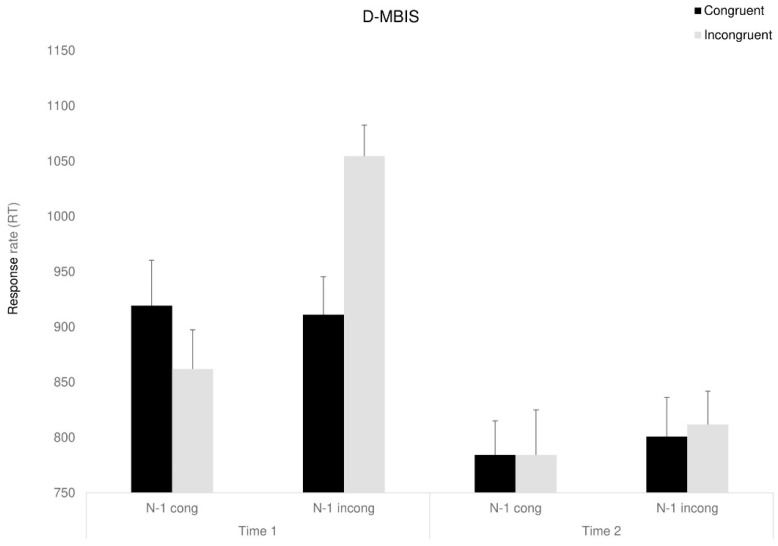
Congruency RT of D-MBIS group as a function of the previous trial (previous congruent: N-1 congruent/previous incongruent: N-1 incongruent) and time. Means (*SD*). Error bars represent standard error from the mean.

**Figure 6:**
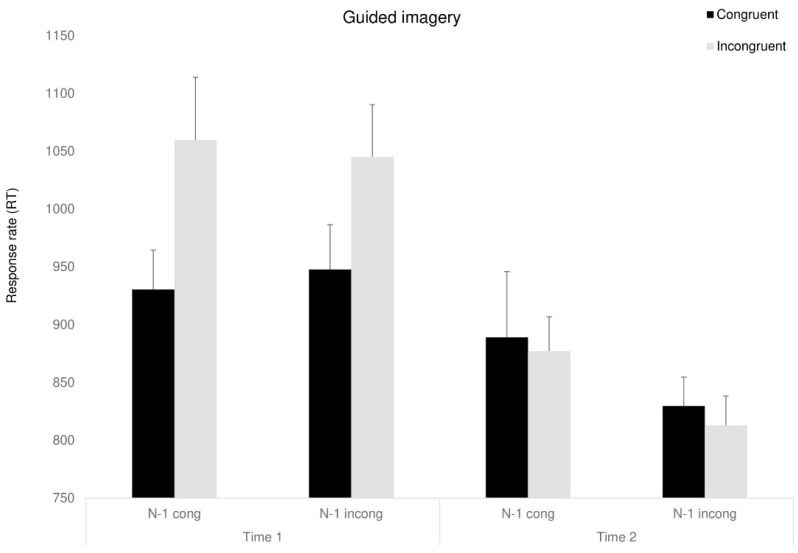
Congruency RT of guided imagery group as a function of the previous trial (previous congruent: N-1 congruent/previous incongruent: N-1 incongruent) and time. Means (*SD*). Error bars represent standard error from the mean.

**Figure 7:**
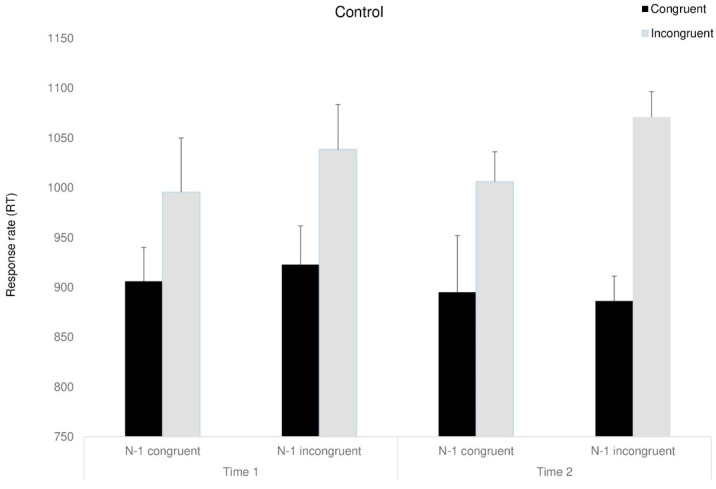
Congruency RT of the control group as a function of the previous trial (previous congruent: N-1 congruent/previous incongruent: N-1 incongruent) and time. Means (*SD*). Error bars represent standard error from the mean.

*F*_(1,8)_ = 7.31, *p* < 0.053, ηp^2^ = 0.47. The four-way Time X Congruency X Previous trial X Group interaction was marginally significant, *F*_(1,8)_ = 3.56, *p* < 0.059, ηp^2^ = 0.30 (see Figure 5, Figure 6 and Figure 7). Further analyses showed that surprisingly, in the D-MBIS group before the intervention (Time 1), the Simon effect appeared after the incongruent trials, *F*_(1,8)_ = 5.65, *p* < 0.054, ηp^2^ = 0.41, but not after the congruent trials, *F*_(1,8)_ < 1. In the guided imagery group before the intervention (Time 1), the Simon effect after both congruent and incongruent trials was marginally significant, *F*_(1, 8)_ = 4.1, *p* < 0.057, ηp^2^ = 0.34, replicating Aisenberg et al.’s (2014) findings. At Time 2, after both interventions, no Simon effect was observed, neither after the congruent nor the incongruent trials. All other effects were not significant (see Table S3 in the Supplementary Materials).

## 4. Discussion

Our study examined the effects of two interventions—a pilot decentering-focused program (D-MBIS) and an equivalent guided imagery intervention—on mindfulness levels, psychological well-being, mental distress, and cognitive abilities among older adults. In doing so, we address the growing interest in developing brief, targeted mindfulness interventions suitable for older populations, particularly given the mixed findings reported for such interventions (e.g., Baranski, 2021; Jankowski & Holas, 2020; Bergen-Cico et al., 2013; Somaraju et al., 2023). We sought to identify effective mechanisms within mindfulness, specifically decentering, that could be feasibly implemented in the community. The study included two group interventions of eight weekly sessions and a control group. This suggests that even brief interventions, which are more accessible to older adults, can cultivate certain mindfulness attitudes (Josefsson et al., 2011; Mackenzie et al., 2006).

### 4.1. Change in Mindfulness Level

An examination of mindfulness levels after the interventions revealed a change in mindfulness skills (FFMQ scores). An increase in the participants’ nonjudgmental attitude was observed in both intervention groups. This finding can be explained by understanding the unique characteristics of older adults, which contributed to their nonjudgmental improvement regardless of manipulation type. Older adults tend to make judgments about themselves as they observe others (Feyers et al., 2010). Therefore, any intervention that involves observations on the self or others could result in more self-empathizing perceptions among seniors. Further support for this explanation is given by when older individuals tended to engage more socioemotional brain networks than younger adults when making self-judgments (Feyers et al., 2010). In other words, it may be that a meeting-group format that involves observing others additively influenced the non-judgment measure beyond the interventions’ content.

Also, participants surprisingly showed an increase in the non-react measure, a variable expected to decrease upon acquiring mindfulness skills. In general, the non-react measure has been robustly associated with well-being (e.g., Baer et al., 2008; Josefsson et al., 2011), but in trying to understand this finding, we again consider the specificity of the senior population. Here, mental non-reaction among seniors may be interpreted as not being able to react, even if desiring to do so. It is likely that the guided imagery intervention, which contained a theme of climbing a mountain, step by step, facilitated visualizing a relatively wide array of details and may have triggered participants’ active processing of thoughts and experiences. This elaborate and active thinking may lead to greater responsiveness, as seen in the decreased non-react measure. In line with this reasoning, the increased reaction to present events seems beneficial for seniors, as this would allow them to perceive changes in their surroundings and address them, thus overcoming age-related challenges and declines. This effect may have emerged in this population as a sense of engagement, reflected in the pursuit of continued stimulation, considered a prominent aspect of successful aging (Reichstadt et al., 2007).

### 4.2. Change in Psychological Distress

As for psychological change, a significant decrease in depression levels (PHQ-9) was observed in both intervention groups but not in the control group. Furthermore, a significant increase in positive relations (PWB) was found in both intervention groups. As we predicted, it appears that our intervention groups facilitated relating to feelings and emotions and hence, reduced depressive symptoms, as in emotionally focused therapy (Soltani et al., 2014). No additional benefit was observed for reducing depression symptoms for the D-MBIS group than for the guided imagery group. This lack of differentiation can be explained by noting that MBSR tends to be more effective for people with severe depression symptoms than among those experiencing minor depression (M. O’Connor et al., 2014), as in our study. Beyond the explanations provided in the previous section (cohort tendency, positive group effect), another factor may account specifically for mood improvement in both groups: choosing to commit to a setting of group meetings of eight weeks may decrease depression symptoms, such as in behavioral activation (Yon & Scogin, 2008). These findings align with previous research on short mindfulness interventions demonstrating benefits for emotional well-being (Cavanagh et al., 2013; Chen et al., 2013). Furthermore, belonging to a social group, creating personal relationships, or creating a meaningful framework of doing something for oneself may also contribute to emotional improvement (Cacioppo et al., 2006).

### 4.3. Change in Cognitive Status

Cognitive status before and after interventions was assessed using the Simon task. The assessment revealed a decrease in response time in time 2, following the end of interventions, among all three groups. This learning effect is expected, as participants performed the same task twice. Furthermore, at time 1 participants were slower to respond following incongruent trials. This trend disappeared after the interventions, but only for the intervention groups. Since older adults show difficulty adjusting in terms of response time, in line with the congruency effect (Aisenberg et al., 2014; Schmitt et al., 2014), it is apparent that the interventions improved cognitive-control processes, specifically the ability to recover following actions that require conflict resolution (Aisenberg et al., 2018). This is also supported by findings showing a significant improvement in accuracy rate specifically for incongruent trials following the interventions, a pattern not evident among the control participants. Increased accuracy, together with a decreased RT, refutes the speed-accuracy trade-off assumption, providing even stronger support for the interventions’ effect in improving cognitive control.

A surprising finding was revealed in the D-MBIS group regarding the Simon effect. Whereas before the D-MBIS intervention (Time 1), the Simon effect was not observed after congruent trials, it did appear after the incongruent trials. However, in the guided imagery group, the Simon effect was observed after both the congruent and incongruent trials. That is, the guided imagery group (and control group) showed an expected pattern of results for their age group (replicating results of Aisenberg et al., 2014). The absence of the Simon effect after congruent trials in the D-MBIS group would be understandable in the presence of very slow RT (Salzer et al., 2014; De Jong et al., 1994), but this was not the case in our results. Thus, this finding requires further inquiry.

### 4.4. Broader Contributions, Limitations and Future Directions

By focusing on decentering as a core mechanism of mindfulness (Fresco et al., 2007; Hölzel et al., 2011), this study contributes to the understanding of how specific mindfulness components might drive therapeutic outcomes in older adults. Our preliminary findings suggest that brief, decentering-oriented sessions can yield cognitive and emotional benefits, complementing evidence that short mindfulness practices are viable even though prior results have been mixed (Schumer et al., 2018; Somaraju et al., 2023). Nonetheless, this study has several limitations. Two limitations relate to our sample: first, the intervention groups comprised a small number of participants. The small sample affected statistical power for several measured effects, showing marginally significant or small effect sizes. A larger sample would likely have been more effective in revealing some of the actual effects. However, a low number of participants in a group was necessary to obtain significant group effects. Thus, future studies using larger sample sizes should include more intervention groups rather than more participants in each group. Controlling for gender effects is also recommended, as our sample involved mainly women and may, therefore, be biased. Additionally, cultural differences may have influenced participant engagement with mindfulness-based interventions. Given that mindfulness practices are deeply rooted in Eastern traditions (Kabat-Zinn, 1990), variations in cultural attitudes toward mindfulness may have shaped participants’ receptivity and responses (e.g., Du & Ning, 2024). Future studies should consider how cultural background moderates intervention effectiveness. Controlling for gender effects is also recommended, as our sample involved mainly women and may therefore be biased. Gender differences in mindfulness engagement have been noted in previous research (e.g., Upchurch & Johnson, 2019), suggesting that men and women may derive different benefits from mindfulness practices. Future studies should aim for a more balanced gender representation to clarify whether observed effects are generalizable across sexes. Moreover, geographic constraints prevented a fully random assignment of participants to the control condition, which may have introduced selection bias. As such, any causal interpretations regarding intervention efficacy should be made with caution. Future studies using stratified or block randomization methods would help confirm the direction of any observed effects.

Other limitations relate to our specific manipulation. First, although homework instructions were matched in both interventions, their performance was not meticulously executed. Limiting the group sessions to 20 min did not allow the instructor sufficient time to examine the quality of home performance. Hence, it is reasonable to assume that considerable variability existed in both quantity and quality of homework completion. Second, we deliberately matched the sessions’ content between the two intervention groups, perhaps causing some overlap, thus diminishing the distinctions between the two processes. This overlap may account for our findings in that almost all the effects were comparable for both intervention groups, making it difficult to ascertain their distinctive contributions. The practical conclusion regarding their effectiveness remains strong and promising, but the scientific understanding of underlying mechanisms still needs further examination.

Another key limitation is that we did not account for individual differences in mindful personality traits, which could have influenced intervention outcomes. Prior research (Rau & Williams, 2016) suggests that dispositional mindfulness varies across individuals and affects the extent to which they benefit from mindfulness-based interventions. Future studies should consider measuring baseline mindful personality traits to determine whether pre-existing tendencies toward mindfulness moderate the observed effects.

The fact that decentering levels were not directly measured made it unclear whether and how we manipulated this component. Future investigations should thoroughly examine the mechanism of change underlying the decentering process and seek to capture the various mindfulness components’ unique respective contributions.

Future studies should consider several essential issues. First, it would be useful to assess long-term benefits at least six months following the program. Second, examining possible individual trait mediators may help identify subpopulations that may be more likely to benefit from a D-MBIS intervention. Additional factors of interest to be examined include the intervention’s effect on older clinical populations (e.g., effects on depression and anxiety) and the impact of more demographic measures. Finally, given that the overall sample was small, and some results were marginal, these conclusions should be viewed as preliminary until replicated with larger, more diverse samples.

## 5. Conclusions

The present study is a promising pilot for a decentering-focused intervention suitable for older adults and an equivalent guided imagery intervention. It contributes to the continuing discourse regarding the agents of change underlying MBSR training. Our brief interventions were associated with improvements in cognitive control and well-being among older adults and appear suitable for an older population. This study’s strengths included administering a comprehensive evaluation, incorporating various psychological measures, as well as an experimental cognitive assessment. Furthermore, two matching interventions were designed alongside a control group. However, additional intervention groups need to be examined to confirm the intervention’s effects, perhaps even specifically in times of social isolation for older adults, that limits their accessibility to other treatment options. In sum, although our pilot interventions showed promising patterns of improvement, several effects did not reach robust statistical significance, underscoring the need for replication with larger and more heterogeneous samples. Our study has immediate implications, as following COVID-19 crisis, it became necessary to validate relevant interventions for seniors who cannot leave their home or facility. Short MBIS and matched guided-imagery intervention can clearly provide such a solution.

## Figures and Tables

**Table 1:** Demographic variables by group.

	Guided Imagery	D-MBIS	Control Group	*N*	*df*	Parameter	*p*-Value
*n* (women)	7 (6)	7 (6)	16 (11)	30 (23)			
Age	76.4 * (5.5) **	73.6 (4.3)	74.5 (3.8)	14	2.27	*F* < 1	n.s.
Education	15.9 (2.9)	14.7 (3.0)	14.4 (3.0)	14	2.27	*F* < 1	n.s.
Unattended Sessions	1.57 (0.98)	1.86 (1.21)	-	14	1.27	*t* = 0.49	n.s.
Language	all reported the maximum level (5), reflecting mother-tongue level						

Note. * Mean; ** (*SD*). n.s. = not significant.

## Data Availability

The data presented in this study are available on request from the corresponding author.

## Supplementary Materials

The following supporting information can be downloaded at: https://www.mdpi.com/article/10.3390/bs15040466/s1, File S1. D-MBIS protocol; Figure S1. Consort flow diagram; Table S1. Guided Imagery protocol; Table S2. Means (and Standard Deviations) of Study Measures Before (Time 1) and After (Time 2) the Interventions; Table S3. Main Effects and Interactions in the Simon Task for Accuracy and Response Time (RT).
