# Supplementary material for: Brief Mindfulness-Based Intervention for Seniors—An Exploratory Semi-Randomized Examination of Decentering Effects on Cognitive Functions and Psychological Distress
Source: Behav Sci (Basel). 2025 Apr 3;15(4):466. doi: 10.3390/bs15040466 (PMC12024281; doi:10.3390/bs15040466)
Supplement: Supplementary file 1 [file behavsci-15-00466-s001.zip › File S1. D-MBIS protocol.pdf]

D-MBIS Group

Mountain Meditation

Hello everyone, how are you? As a reminder, we will meet on this day and time each week for the next eight weeks for sessions of about 20 minutes. Between meetings, you'll be given home assignments, which I'll ask you to bring to the sessions. At the end of the eight weekly sessions, I'll meet with each of you for an additional personal meeting, which we'll schedule later.

Each of our shared practices will consist of about 15 minutes of meditation, followed by 5 minutes of open discussion where I'll be happy to hear about your experience with the practice and the assignments.

Any questions so far?

In the next 20 minutes, we'll practice the meditation together. Please sit comfortably in your chairs. I'll guide you through the practice, starting with the sound of the gong. I'll ring it again to signal the end of the session.

Sit in a comfortable position that doesn't require much effort.
Sit upright—but not too rigid, so you don't get tired. Not too relaxed either, so you don't fall asleep. You can imagine a golden thread pulling you upward, from the point of connection with the chair up through the crown of your head. Not too tight, not too loose—just right.
Rest your hands in a comfortable, relaxed position that allows you to stay still.
Gently close your eyes. If closing your eyes doesn't feel comfortable, you can fix your gaze softly on a point in front of you.

Gong

Let's take three deep breaths.
Now, as you return to a natural rhythm of breathing, focus on your breath—each inhale and exhale. Notice how the air enters and exits the body.
Observe the pace: Is it even? Is your inhale longer than your exhale, or the opposite?

We're not trying to control the breath—just observe it as it is.
Let it be, as it is in this moment.
The breath is our anchor to the present.

During the practice, whenever you notice your mind has wandered, simply acknowledge it and gently guide your attention back to the breath. When you're ready, allow your attention to soften.
Now, open your awareness to everything occurring right now.

Imagine yourself as a mountain—exposed to all elements.
Your head and shoulders are the peak, your legs rooted deep in the ground.

The mountain doesn’t move; it doesn’t react to wind, rain, or sun.
It is part of nature—quiet and steady.

Animals climb the mountain and descend, the mountain receives them—lets them come, lets them go. Rain falls and stops. The sun rises and sets. We too sit in stability, allowing all thoughts and sensations to come and go.

A sensation arises—just notice it gently.
Recognize it but don’t go deeper right now—return to the breath.

A memory or image might surface—just acknowledge it.
Be the mountain: notice, don’t interpret or judge.

A thought arises—“a thought about this or that.”
Let it pass like a cloud drifting in the sky.
The mountain remains still—present—breathing.

A feeling may come.
Treat it like internal weather.
A storm may come and go.
A heatwave may last long—but the mountain doesn’t resist—it waits with patience.

If a particular thought emerges—welcome it gently, without judgment.
Return softly to your anchor—your natural, calm breath.

Inhale. Exhale.

What am I focused on now?
Whatever it is—I can always come back to the breath. To this moment.

We observe ourselves as part of nature, where every event is fleeting.
Inhale, exhale—again and again. This is the present moment.

The mountain breathes naturally.
It welcomes nature's occurrences and remains steady.

A sound, a smell, a taste, a thought, a feeling—these are all natural phenomena.
The mountain witnesses them.

No event is good or bad. No feeling right or wrong.
All are equal and transient.

Let’s sit a few more minutes in silence—steady as a mountain—until you hear the gong.

Inhale. Exhale.

Gong

Homework

Sit for a few minutes each day using the guidance given in the session. You may repeat key ideas—imagining the mountain, noticing thoughts as passing, returning to the breath, etc. Write down anything that comes up during the practice and bring it to the next meeting.

Lake Meditation

Let's make sure we are seated in a comfortable posture, one that does not require much effort. We'll aim to sit upright, but not too upright so as not to become fatigued. And not too relaxed, to avoid falling asleep. Imagine a golden thread gently pulling you upward—from the point where your body meets the chair, up through the crown of your head. Not too tight, not too loose—just right.

Once you're settled, we'll begin the meditation. Take three deep breaths. Then, as we return to our natural, regular breath, try to focus on the physical sensation of breathing. We're not trying to change the breath, just to observe it—simply and naturally. Let it rest. The breath is our anchor to the here and now, to the present moment. In this practice, every time the mind wanders to another thought or sensation, we gently acknowledge that the attention has wandered and return, softly and kindly, to the feeling of the breath.

When you feel comfortable and ready, allow your attention to relax from the breath.
Now, open your awareness to whatever is present in the moment.

And as you are right now, imagine in your mind's eye a picture of a lake—a body of water, large or small, resting in the embracing ground of the Earth itself.
Notice how water naturally seeps, flows, and gathers in low places, seeking its proper level, wanting to be held and contained.

The lake might be deep or shallow, blue or green, murky or clear.
Without wind, the surface is still, like a mirror, reflecting trees, rocks, clouds in the sky—holding it all, moment by moment.

A breeze may come and create ripples on the water, distorting or erasing the reflections—but then a ray of light may sparkle and dance across the tiny waves, like shining diamonds…

When night arrives, the moon takes its turn to dance on the lake…
Or when the surface is still, the outlines of trees and shadows are reflected peacefully.
In winter, the lake may freeze—but still, underneath, it brims with movement and life.

As you rest with your breath and the image of the lake becomes clearer in your awareness, allow yourself—when you're ready—to fully bring the image of the lake into yourself, so you and the lake become one.

Your entire energy is now held in awareness—with openness and compassion for yourself—just as the lake is held by the Earth's embracing ground.

Breathe like the lake.
Feel the waters of the lake as your own body.
Let your mind and heart be open and accepting, moment by moment.
Allow reflection of all that surrounds you—or just remain pure and clear to the depths.
Feel moments of perfect calm, when both the reflections and waters are crystal clear.
And in other moments, when the surface becomes wavy, disturbed, murky—the reflections and depth may momentarily disappear.

And amidst it all, you sit here—simply observing the shifting phenomena of your mind and heart: passing thoughts and emotions, urges and reactions—rising and falling like ripples or waves.
Notice how they affect you, just as you would observe the changing surface of a lake—wind, waves, glimmering light, shadows, reflections, colors, and scents.

Be aware of how your thoughts and emotions impact you.
Do they disrupt the stillness and clarity of your mind-lake?
Do they make the water murkier?
Is that okay with you?
Allow yourself to simply observe—how thoughts, emotions, and sensations influence your inner lake.
And with compassion and gentleness, notice how you respond to those changes.

Are the waves and fluctuations in the water not part of simply being a lake?
Can you identify not only with the surface of your inner lake, but also with its entire body of water—so that you also become the calm and stillness beneath, which usually only feels gentle ripples, even when the surface is stormy?

In this same way, during home practice and in daily life, can you connect not only with the changing content and intensity of your thoughts and feelings, but also with the vast and stable reservoir of awareness beneath the surface of your consciousness?
The lake can teach us this.
It reminds us of the lake within us.

Be present here in the stillness of this moment.
Until the sound of the bell, we can be the silent lake, recognizing our capacity to remain in awareness and acceptance.
In this moment, all the qualities of our body and mind are held—just as the lake is cradled by the earth—reflecting sun, moon, stars, trees, clouds, birds, and light, caressed by the wind and air, which bring out its sparkle, vitality, and potential—moment by moment.

So in the remaining time, until the bell signals the end of this meditation, continue to carry the image of the lake within yourself, in silence, moment by moment—be the lake with its moments of storm and serenity alike.
