# Supplementary material for: Brief Mindfulness-Based Intervention for Seniors—An Exploratory Semi-Randomized Examination of Decentering Effects on Cognitive Functions and Psychological Distress
Source: Behav Sci (Basel). 2025 Apr 3;15(4):466. doi: 10.3390/bs15040466 (PMC12024281; doi:10.3390/bs15040466)
Supplement: Supplementary file 1 [file behavsci-15-00466-s001.zip › Table S1. Guided Imagery protocol.pdf]

D-MBIS Group

Mountain Meditation

Hello everyone, how are you? As a reminder, we will meet on this day and time each week for the next eight weeks for sessions of about 20 minutes. Between meetings, you’ll be given home assignments, which I’ll ask you to bring to the sessions. At the end of the eight weekly sessions, I’ll meet with each of you for an additional personal meeting, which we’ll schedule later.

Each of our shared practices will consist of about 15 minutes of meditation, followed by 5 minutes of open discussion where I’ll be happy to hear about your experience with the practice and the assignments.

Any questions so far?

In the next 20 minutes, we’ll practice the meditation together. Please sit comfortably in your chairs. I’ll guide you through the practice, starting with the sound of the gong. I’ll ring it again to signal the end of the session.

Make sure to sit in a comfortable position, one that doesn’t require much effort. Sit upright, but not too rigidly, so as not to become tired. And not too relaxed, to avoid falling asleep.
Imagine a golden thread gently pulling you upward—from where your body touches the chair, through the crown of your head. Not too tight, not too loose—just right. Once you're settled, we’ll begin.

Close your eyes and count 10 natural breaths.
You don’t need to deepen or extend them—just breathe as you normally would. Simply count and pay attention—notice when air flows in (inhalation) and when it flows out (exhalation). As you count the breaths, you’ll notice your body becoming more and more relaxed.

Here comes the first breath.
And the second.
Already, calmness begins to seep in.
And another breath…
Your body is becoming more and more still… more and more relaxed.
Your overall feeling is growing increasingly peaceful…
More and more at ease.

Now that we’ve entered our comfort zone, picture a hiker climbing a mountain.
The air is crisp and clean, and he breathes in the fresh air of nature.
He looks around and sees a trail winding upward—not too steep, not too gentle.
Our hiker—we’ll call him Natan—starts walking.

Along the way, he gradually notices wildflowers—cyclamens blooming between rocks, dew resting on fragrant lavender bushes.
These scents—sweet smells of spring and renewal—bring back memories… childhood recollections. A smile spreads across his face. He feels good, free in this open space, immersed in nature.

The climb suits him, matching his inner rhythm, and all this peace—inner and outer—makes his steps light.
Each step taps the ground with a soft rhythm, like music accompanying him wherever he goes.

Inhale… exhale…
At this point, take a few more breaths… bringing fresh oxygen into the body.
With each inhale, we draw in fresh, oxygen-rich air.
The fresh air revitalizes our tissues, calms the body, and brings a sense of ease and serenity.

Natan reaches the mountain peak.
Filled with pride, he looks around at the breathtaking view—open skies, endless fields, stillness, and infinite space.
This image of him slowly fades in our minds—we see him from above, his figure receding into the horizon.
Let's set Natan aside for now.

Now, as we near the end, let's release with each breath the tensions that were previously within us. Continue with about 20 more breaths like this.
Inhale… exhale…
We feel cleaner, more peaceful, freer.
Stillness seeps into us.
And the body becomes quieter and quieter…

Before ending the session, let's remain in silence and take a few more breaths at our own pace, until the gong sounds again.
When the sound is heard, each person may open their eyes in their own time.
Inhale… exhale…

GONG

Homework Assignments:

Practice daily each week—throughout the entire course.

Lake Meditation

Let's make sure we are seated in a comfortable position, one that doesn't require much effort. Sit upright, but not too upright so as not to become tired. And not too relaxed, so you don't fall asleep. Imagine a golden thread gently pulling you upward—from the point of contact with the

chair, through the crown of your head. Not too tight, not too loose—just right.
Once we are settled, we will begin the meditation.

Close your eyes, and count ten natural breaths.
There's no need to do anything special with these breaths—no need to deepen or extend them.
Just ten breaths at your current natural rhythm. The only difference is that you're counting them and noticing: when the air comes in (inhalation) and when it goes out (exhalation).
As we count, we'll begin to notice our body growing more and more relaxed.

Here is the first breath.
And the second.
Already, stillness begins to seep into us.
And another breath...
The body becomes calmer... more and more relaxed.
The overall feeling becomes more and more quiet, more and more peaceful.
More and more at ease.

Now that we've entered our comfort zone, and just as you are right now, imagine in your mind's eye an image of a lake—a body of water, large or small, resting in the containing ground of the earth. Notice how water tends to seep, to flow, to gather in low places, seeking its own level, longing to be held and contained.

Near the lake sits a traveler, a passerby. Let's call her Hila.
She is seated on the ground, close to the lakeshore, leaning against a large and sturdy tree.
Her legs are crossed one over the other, and she gazes calmly at the lake.
She notices the blue-green shades of the water. She sees how the calm surface reflects the trees, rocks, and clouds in the sky—holding it all, moment by moment.

Suddenly, a gentle breeze stirs Hila's hair, and she notices ripples forming in the lake...
The reflections shift and sway with the small waves... And between the gentle wave motions, a single clear beam of light appears—sparkling and dancing among the small waves. To Hila, this image looks like shimmering diamonds...

Inhale... exhale...
At this point, let's take a few more breaths... bringing oxygen into the body.
With each inhalation, we draw fresh, oxygen-rich air into the body.
The fresh air rejuvenates the tissues, calms the body, and gives us a sense of peace and ease.

Slowly, evening falls, and now it's the moonlight dancing on the lake...
After a while, as the breeze settles and the water becomes still again, the full moon reflects on the surface... along with the silhouettes of trees and other shadows. Hila watches this breathtaking scene. She is mesmerized and moved by the magical beauty of nature.

Now this image of her begins to slowly drift away in our minds—we see her from a bird's-eye view, her figure retreating into the distance.
Let us now release Hila from our awareness.

As we approach the end, with each breath, let us release the tension that was in us earlier.
Let's continue with about 20 more breaths like this.
Inhale... exhale...
We feel cleaner, more serene, freer.
Stillness seeps into us.
And the body becomes quieter and quieter...

Before we conclude the session, let us remain in silence and take a few more breaths at our own pace, until the sound of the gong returns.
When the sound is heard, each person may open their eyes in their own time.
Inhale... exhale...

GONG

Homework Assignments:

Practice daily every week throughout the course.
