# Supplementary material for: Brief Mindfulness-Based Intervention for Seniors—An Exploratory Semi-Randomized Examination of Decentering Effects on Cognitive Functions and Psychological Distress
Source: Behav Sci (Basel). 2025 Apr 3;15(4):466. doi: 10.3390/bs15040466 (PMC12024281; doi:10.3390/bs15040466)
Supplement: Supplementary file 1 [file behavsci-15-00466-s001.zip › Table S2 and S3.pdf]

Supplemental materials

Participants		Time 1				Time 2			
**Factor**	(*N* =)	GI	D-MBIS	control	total	GI	D-MBIS	control	total
**mental status (MMSE)**	30	28.9 (0.9)	29.6 (0.5)	29 (0.9)	28.8 (0.9)	29.4 (0.8)	29 (1.2)	28.75 (0.9)	28.9 (0.9)
**depression (BDI-II)**	13	11.1 (9.4)	7.3 (4.8)		9.2 (7.4)	10.7 (8.9)	7.0 (3.8)		8.7 (6.6)
**psychological distress (PHQ-9)**	29	9.5 (5.3)	6.1 (4.7)	6.6 (2.9)	6.7 (2.7)	5.7 (2.6)	4.7 (3.8)	6.6 (2.7)	6 (2.9)
**Being Scales (PWB) -Psychological Well**	13	4.0 (0.9)	4.5 (0.5)		4.3 (0.7)	4.0 (0.8)	4.4 (0.4)		4.2 (0.6)
**Autonomy (PWB1)**	13	4.5 (0.9)	4.3 (0.6)		4.4 (0.7)	4.2 (0.7)	4.3 (0.4)		4.3 (0.5)
**Environmental Mastery (PWB2)**	13	3.9 (1.0)	4.6 (0.6)		4.2 (0.9)	4.0 (1.0)	4.5 (0.5)		4.3 (0.8)
**Personal Growth (PWB3)**	13	4.4 (1.1)	4.5 (0.6)		4.4 (0.8)	4.2 (1.0)	4.6 (0.4)		4.4 (0.7)
**Positive Relations (PWB4)**	12	3.6 (0.8)	4.3 (0.9)		4.0 (1.0)	(0.8)4.1	4.6 (0.9)		4.4 (0.9)
**Purpose in Life (PWB5)**	12	4.1 (1.0)	4.7 (0.7)		4.4 (0.9)	4.0 (0.9)	4.3 (0.7)		4.2 (0.8)
**Acceptance (PWB6)-Self**	10	3.7 (1.5)	4.6 (0.6)		4.1 (1.2)	3.6 (1.6)	4.4 (0.8)		4.1 (1.2)
**Mindfulness (FFMQ)**	13	3.4 (0.7)	3.5 (0.5)		3.4 (0.6)	3.4 (0.5)	(0.2)3.5		3.5 (0.3)
**Observation (FFMQ1)**	13	3.4 (0.6)	3.7 (1.1)		3.5 (0.9)	3.5 (0.6)	3.8 (0.6)		3.7 (0.6)
**Describe (FFMQ2)**	13	3.5 (0.9)	3.9 (0.6)		3.7 (0.8)	3.7 (0.6)	3.8 (0.3)		3.8 (0.5)
**Act with Awareness (FFMQ3)**	11	3.5 (0.9)	3.3 (0.6)		3.4 (0.7)	3.1 (0.5)	(0.4)3.4		3.3 (0.5)
**Judgment (FFMQ4) -Non**	10	3.5 (1.2)	2.9 (0.7)		3.2 (1.0)	3.6 (0.9)	3.3 (0.7)		3.4 (0.7)
**React (FFMQ5)-Non**	13	3.2 (0.1)	3.3 (0.6)		3.3 (0.6)	2.9 (0.6)	3.1 (0.5)		3.0 (0.6)

Table S2**.** *Means (and Standard Deviations) of Study Measures Before (Time 1) and After (Time 2) the Interventions.* Note. GI = Guided Imagery; D-MBIS = Decentering Mindfulness-Based Intervention for Seniors; MMSE = Mini-Mental State Examination; BDI-II = Beck Depression Inventory-II; PHQ-9 = Patient Health Questionnaire; PWB = Psychological Well-Being Scale; FFMQ = Five Facet Mindfulness Questionnaire.

**Accuracy effects**	***df***	***F***	***MSE***	ηp^2	***p* <**
group	2,24	2.39	0.0241	.16	*n.s*
time	2,24	1.41	0.0075	.05	*n.s*
time x group	2,24	1.29	0.0075	.09	*n.s*
congruency	2,48	3.43	0.0064	.12	.04
congruency x group	4,48	2.06	0.0064	.14	*n.s*
time x congruency	4,48	2.64	0.0039	.09	*n.s*
time x group x congruency	4,48	2.06	0.0039	.14	*n.s*
**RT effects**	***df***	***F***	***MSE***		***p* <**
group	2,23	<1	202693	.05	*n.s*
time	2,23	5.07	64249	.18	.057
time x group	2,23	1.84	64249	.13	*n.s*
congruency	2,23	8.2	20419	.26	.008
congruency x group	2,23	2.25	20419	.16	*n.s*
time x congruency	2,23	1.47	9686	.06	*n.s*
time x group x congruency	2,23	3.47	9686	.23	.048
greater improvement for intervention groups	1,23	7.31			.003
previous	2,23	<1	18435	.02	*n.s*
previous x group	2,23	1.08	18435	.08	*n.s*
previous x congruency	2,23	<1	22307	.02	*n.s*
previous x time	2,23	3.12	7430	.11	*n.s*
previous x group x time	2,23	1.03	7430	.08	*n.s*
previous x group x congruency	2,23	<1	22307	.02	*n.s*
previous x congruency x time	2,23	<1	31530	.01	*n.s*
previous x group (without control) x congruency x time	2,23	3.56	47551	.31	.095
Simon effect following incongruent but not following congruent, for D-MBIS group on time 1	2.23	5.65			.044

Table S3. *Main Effects and Interactions in the Simon Task for Accuracy and Response Time (RT).*
Note. GI = Guided Imagery; D-MBIS = Decentering Mindfulness-Based Intervention for Seniors; RT = Response Time; *MSE* = Mean Square Error; ηp^2 = Partial Eta Squared.
